# Should women with chronic pelvic pain have adhesiolysis?

**DOI:** 10.1186/1472-6874-14-36

**Published:** 2014-03-04

**Authors:** Ying C Cheong, Isobel Reading, Sarah Bailey, Khaled Sadek, William Ledger, Tin C Li

**Affiliations:** 1 Human Development and Health, Faculty of Medicine, University of Southampton, Coxford Road, Southampton SO16 5YA, UK; 2 Complete Fertility Centre Southampton, Princess Anne Hospital, Coxford Road, SO16 5YA Southampton, UK; 3 Royal Hallamshire Hospital, Jessop Wing, Tree Root Walk, S10 2 SF Sheffield, UK; 4 University of New South Wales, UNSW, Sydney, NSW 2052, Australia; 5 Research Design Service, Southampton General Hospital, Coxford Road, SO16 5YA Southampton, UK

**Keywords:** Chronic pelvic pain, Adhesiolysis, Surgery, Laparoscopy, Randomised controlled trial

## Abstract

**Background:**

Pelvic adhesions are found in up to 50% of women with CPP during investigative surgeries and adhesiolysis is often performed as part of their management although the causal or casual association of adhesions, and the clinical benefit of adhesiolysis in the context of CPP is still unclear. Our aim was to test the hypothesis of whether laparoscopic adhesiolysis leads to significant pain relief and improvement in quality of life (QoL) in patients with chronic pelvic pain (CPP) and adhesions.

**Methods:**

This was a double-blinded RCT. This study was conducted in 2 tertiary referral hospitals in United Kingdom over 4 years. Women with chronic pelvic pain (CPP) were randomized into having laparoscopic adhesiolysis or diagnostic laparoscopy. Women were assessed at 0, 3 and 6 months for Visual analogue scale scores (VAS) and Quality of Life (QoL) measures (SF-12 and EHP-30).

**Results:**

A total of 92 participants were recruited; 50 qualified to be randomized, with 26 in the adhesiolysis and 24 in the control group. The results are expressed in median (interquartile ranges). In women who underwent adhesiolysis, there was a significant improvement at 6 months in VAS scores (-17.5 (-36.0 - -5.0) compared to controls (-1.5 (-15.0 – 4.5; p = 0.048); SF-12 scores physical component score (25.0 (18.8 – 43.8)) compared to controls (6.3 (-6.3 – 18.8); p = 0.021), SF-12 emotional component score 32.5 (4.4 – 48.8) compared to controls -5 (-21.3 – 15.0); p < 0.0074) and EHP-30 emotional well being domain 32.5 (4.4 – 48.8) compared to the controls -5 (-21.3 – 15.0; p < 0.0074).

**Conclusions:**

This study stopped before recruitment reached the statistically powered sample size due to difficulty with enrollment and lack of continued funding. In selected population of women presenting to the gynecological clinic with chronic pelvic pain, adhesiolysis in those who have adhesions may be of benefit in terms of improvement of pain and their quality of life.

**Trial registration number:**

ISRCTN 43852269

## Background

Chronic pelvic pain (CPP) is a debilitating condition with a heterogeneous aetiology and a high disease burden worldwide [1]. CPP has a reported incidence in primary care of 38 per 1000 women, which is comparable to the incidence of back pain (41 per 1000) and asthma (37 per 1000) [2,3]. Approximately 20% of all outpatient appointments in secondary care is attributed to CPP [4]. Up to £158 million (UK) and $881 million (US) health care resources is spent managing CPP annually, and Maastricht these are likely to be underestimates [5,6]. Pelvic adhesions are found in up to 50% of women with CPP during investigative surgeries [7-9] and adhesiolysis is often performed as part of their management although the causal or casual association of adhesions, and the clinical benefit of adhesiolysis in the context of CPP is still unclear and controversial [10,11].

Only two randomized controlled trial has evaluated the role of adhesiolysis in pain [12,13]. The first study by Peters et al reported that adhesiolysis may possibly benefit women with more severe adhesions involving the gastrointestinal tract but the study was performed on women undergoing adhesiolysis during laparotomy, a procedure known to be more adhesiogenic then laparoscopy and had a small sample size (n = 48). Swank et al. [13] examined the effectiveness of laparoscopic adhesiolysis on patients with chronic abdominal pain in both men and women and reported that although laparoscopic adhesiolysis relieves chronic abdominal pain, it was no better than diagnostic laparoscopy. But the study was not specifically design to examine CPP and did not report on the condition of the pelvis and hence, their results cannot be extrapolated directly to women with CPP. The Cochrane review stated; “there is still uncertainty about the place of adhesiolysis among patients presenting to gynaecologists” and that there is “no evidence of benefit” rather than ’evidence of no benefit’. The review recommended that further large trials of adhesiolysis recruiting ‘gynaecological’ patients to provide the necessary level of evidence [10].

This study was therefore designed with the objective to evaluate the hypothesis that laparoscopic adhesiolysis leads to significant pain relief and improvement in quality of life (QoL) in patients with CPP and adhesions in order to provide scientific basis for sound clinical practice which in turn can benefit patients who suffer from this distressing condition.

## Methods

This was a two-center study performed in the Princess Anne Hospital, Southampton University Hospitals NHS Trust and Jessop Wing, Sheffield University Hospital Trusts, UK. It was a double-blinded randomised controlled trial where the participants were randomised to either undergo laparoscopic adhesiolysis or diagnostic laparoscopy. Prior to surgery, standard demographic details were obtained and surgical details were obtained during the surgery including the adhesion site, severity and extent in accordance to a standard adhesion scoring system. The follow up intervals were 3, 6, and an optional 12 months after surgery. At the initial and subsequent follow up visits, the following outcome measures were obtained: Pain scores: VAS (visual analogue score) from the McGill pain questionnaire (a self-report questionnaire for intensity and quality of pain) [14], SF-12 (medical outcomes study with 12 item short-form health survey – a short generic measure of subjective health status which includes 12 items encompassing the self assessment of health, physical functioning, physical role limitation, mental role limitation, social functioning, mental health and pain) [15] and modified EHP-30 (endometriosis health profile) questionnaire for pelvic pain [16]. The EHP-30 is a health related quality of life patient self-report, used to measure the wide range of effects of endometriosis. It consists of core instruments on five scale scores, namely pain, control and awareness, social support, emotional well-being and self-image.

The eligibility criteria were pre-operatively, criteria for inclusion: 1) women age over 18 years old; 2) presence of chronic pelvic pain defined as pelvic pain which is constant/cyclical in nature for greater or equal to 6 months duration; 3) written consent; criteria for exclusion: 1) malignancy; 2) psychiatric disorders for which the patient is taking medication; 3) pathology which requires urgent treatment, such as ovarian cyst or pelvic abscess; 4) women taking central nervous system stimulants and 5) pregnancy. The intraoperative criteria were: 1) non-endometriosis related adhesions detected at laparoscopy (determined by gross inspection) and 2) the nature and extent of adhesions considered suitable for laparoscopic adhesiolysis (not via laparotomy). Patients are screened for inclusion of the study using the pre-operative eligibility criteria. Once the preoperative criteria were satisfied, and the laparoscopy begun, the intraoperative criteria were then determined.

Patients were randomised to have either laparoscopic adhesiolysis or diagnostic laparoscopy. Randomisation was performed using a computer generated random numbers and the concealed, opaque unlabeled envelope was opened after it had been determined that the patient met the intraoperative criteria. The patients were blinded to the allocation of treatment and the assessor during follow up was blinded to the treatment. The assessor who administered the questionnaires and recruited the patients was the research nurse who did not have prior knowledge of what type of surgery the patients underwent. Consent was obtained prior to any baseline assessments. The operation notes were stored in a sealed enveloped within the patient notes and not accessed except during an emergency. In the latter case, the data would be used to the point of unblinding. The randomization code was broken at the end of the follow up period and patients who wished to know were informed of their treatment groups. Laparoscopic surgeons who were skilled in advanced laparoscopy performed the surgery. Entry into the abdomen was either via the traditional Veress needle or a modified Hasson’s technique of open entry. CO_2_ was used for creating a pneumo-peritoneum of 20 mmHg before a 10 mm trocar was inserted into the intra-umbilical incision. Two or three more lateral ports were inserted depending on the site and extent of surgery. During surgery, the principles of microsurgery were followed, including meticulous haemostatic control and usage of constant irrigation to prevent tissue desiccation. One litre of 4% Icodextrin (Adept, Baxter) was instilled intraperitoneally after surgery, in the study as well as the control group.

The patients’ histories, clinical examination and operative findings were documented on standard proforma. The extent, severity and site of adhesions was scored using a well-validated adhesion scoring system [17] and the completeness of adhesiolysis was documented. All patients’ data and questionnaires regarding pain and quality of life assessment (VAS, SF-12 and EHP-30) scores were entered into a computerised database. Complications during and after the surgery were documented on standard proforma sheets.

The primary outcome measure was pain score denoted by VAS (visual analogue score) from the McGill pain questionnaire and the secondary outcomes were a) Quality of life scores - SF-12 Health related quality of life scores (medical outcomes study with 12 item short-form health survey); the modified EHP-30 core questionnaire for pelvic pain and b) clinical outcomes - including complications and adverse events.

A sample size of 50 patients per group was needed to detect a difference of 10 points on a 100 point (considered clinically significant [18]) VAS scale between the placebo and active groups with 80% power, using a t-test on the VAS change from baseline, and assuming that the standard deviation for this change is 18.5. Analysis was performed using SPSS (Statistical Package for Social Sciences, V22) on an intention to treat basis regardless of complications or completeness of adhesiolysis. Proportions were compared with the chi-square test. Differences in VAS scores, EHP-30 and SF-12 scores were compared with the Mann Whitney test. For continuous variables, the t-test was used. Within group comparisons was made using the Wilcoxon’s signed ranked test. Due to the distribution of the data, the results are expressed in median and the respective interquartile range (IQR).

The study was reviewed and approved by the South Sheffield Ethics Committee (05/Q2305/159) and Southampton R&D approval from University Hospital Southampton NHS Foundation Trust (ISRCTN 43852269).

## Results

**Figure 1:**
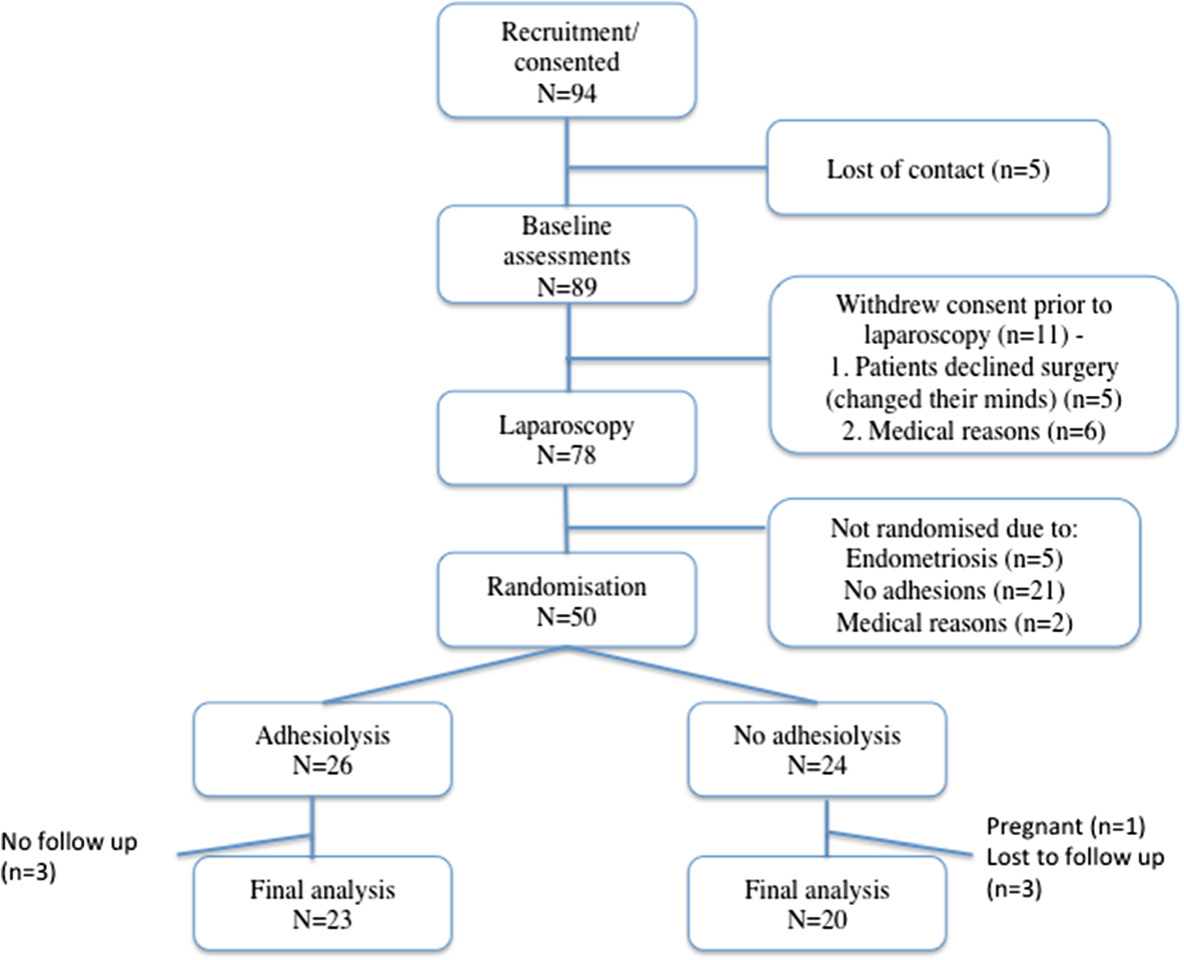
Selection of participants for the laparoscopic adhesiolysis for chronic pelvic pain trial.

Between December 2008 and December 2012, 94 women who satisfied the eligibility criteria for the study were recruited (Figure 1). This study was closed prior to the sample size being reached due to the protracted recruitment period and lack of continued study funding. Five women were loss to contact before the baseline assessments were performed, 11 women withdrew after enrolment prior to laparoscopy: 5 declined surgery (changed their minds about having surgery) after being consented and 6 were withdrawn from the study due to other medical reasons which has arisen after they have been consented (not within the exclusion criteria). 78 women proceeded to have a laparoscopy, of which 21 had no adhesions and therefore not randomised, 5 had endometriosis and were not randomised and 2 withdraw due to other medical reasons not within the exclusion criteria (pregnancy, illness on the day of surgery). 50 women were randomised, n = 26 in the adhesiolysis group and n = 24 in the no adhesiolysis group. Three women had incomplete VAS scores at 6 months. Adhesiolysis was completed in all the women in the study group. None of the participants elected to have a 12-month follow up. Given the small sample size, post-hoc analysis showed that the study now has a 50% power.

Table 1 shows the baseline assessment characteristics of the participants. There was no difference in the baseline assessment characteristics between the study and control group except the adhesion score in the study group (n = 23; median 15 IQR 9 – 29) was higher than controls (median 9, IQR 2.5 – 14; p = 0.034). In other words, women who had adhesiolysis had statistically more severe adhesions than controls.

**Table 1:** Patient characteristics

**Characteristic**	**Treated (N = 23)**	**Control (N = 20)**	**Total (43)**
Age, years (median, IQR)	31 (27 – 46)	30 (26 – 36)	31 (27 – 41)
Length of symptoms, years (median, IQR)	3.2 (1.1 – 8.1)	2.0 (1.1 – 6.7)	3.0 (1.1 – 8.1)
Length of diagnosis, years (median, IQR)	3.1 (1.3 – 8.9)	1.6 (0.8 – 6.6)	2.5 (1.0 – 8.6)
Had one or more previous operation to treat pelvic pain	10 (43)	7 (35)	17 (40)
Adhesion assessment score (median, IQR)	*15 (9 – 29)	*9 (2.5 – 14)	11 (6 – 23)

*P < 0.05.

**Figure 2:**
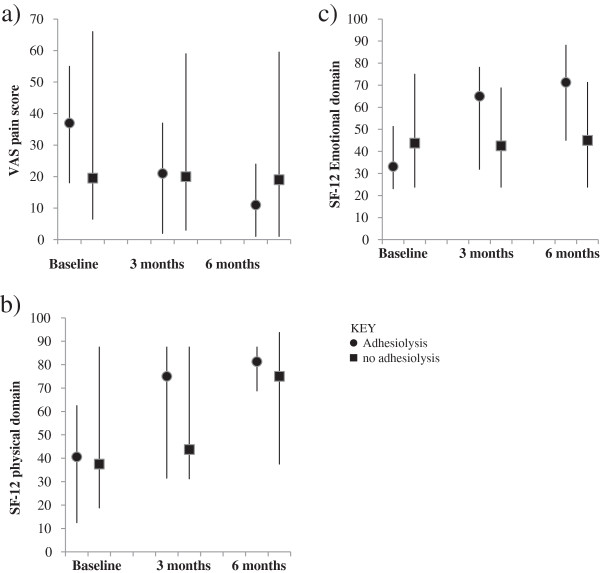
**VAS and SF-12 scores in women with and without adhesiolysis. a** Graph indicates VAS scores in women with adhesiolysis and no adhesiolysis at baseline, 3 months and 6 months. Negative change indicates improvement. 2**b** and **c**. Graph indicates SF-12 (physical and emotional domain) in women with adhesiolysis and no adhesiolysis at baseline, 3 months and 6 months. Positive change indicates improvement. Negative change indicates decline. The bars represent the interquartile range.

**Figure 3:**
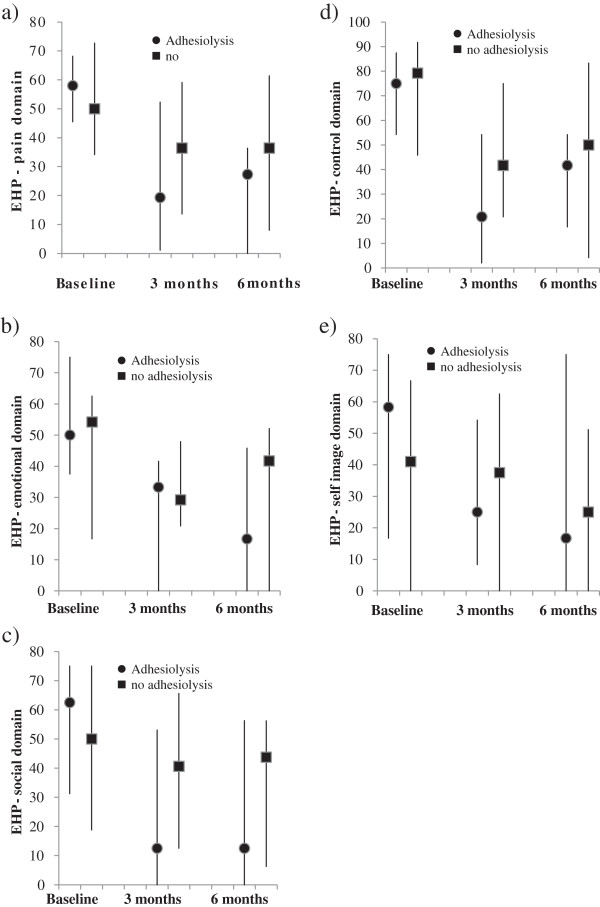
**Graphs indicate EHP-30 (pain, emotional, social, control and self-image domains) in women with adhesiolysis and no adhesiolysis at baseline, 3 months and 6 months.** Positive change indicates decline. Negative change indicates improvement. Bars represent interquartile range. **a**, **b**, **c**, **d**, **e**: circle represents the EHP scores for those who had adhesiolysis, squares represents who had no adhesiolysis.

Figures 2 and 3 illustrate the absolute values of VAS, SF-12 and EHP 30 scores at baseline, 3 and 6 months. Table 2 illustrates the change from baseline data of VAS score, SF-12 and EHP-30 in women who had adhesiolysis compared to those who did not.

**Table 2:** Change from baseline data of VAS score, SF-12 and EHP-30 in women who had adhesiolysis compared to those who did not

**Outcome measures**	**Adhesiolysis (median, IQR)**	**No adhesiolysis (median, IQR)**	**P values**
**VAS scores**			
Change: baseline to 6 months	-17.5 (-36.0 - -5.0)	-1.5 (-15.0 – 4.5)	<0.05
**SF-12 – physical component**			
Change: baseline to 6 months	25.0 (18.8 – 43.8)	6.3 (-6.3 – 18.8)	<0.05
**SF-12 – emotional component**			
Change: baseline to 6 months	32.5 (4.4 – 48.8)	-5 (-21.3 – 15.0)	<0.01
**EHP-30 – emotional well being doman**			
Change: baseline to 6 months	-37.5 (-45.8 - -4.2)	-8.3 (-16.7 – 12.5)	<0.05
**EHP-30 – social support domain**			
Change: baseline to 3 months	-12.5 (-71.9 – 0.0)	-6.25 (-18.8 – 12.5)	<0.05

### VAS

In the treatment group, a general trend of improvement in VAS scores and the SF-12 domains is observed. In women who underwent adhesiolysis, there was a significant improvement in VAS scores at 6 months (-17.5 (-36.0 – -5.0) compared to controls (-1.5 (-15.0 – 4.5); p = 0.048).

### SF-12

In women who underwent adhesiolysis, there was a signficant improvement in the SF-12 physical component score (25.0 (18.8 – 43.8)) compared to controls (6.3 (-6.3 – 18.8); p = 0.021) as well as an improvement in the emotional component score (adhesiolysis: 32.5 (4.4 – 48.8); controls -5 (-21.3 – 15.0); p < 0.0074) at 6 months.

### EHP

Except for the EHP-pain and control domains, all other EHP domains showed a trend of general improvement. There was a signficant improvement in the emotional well being domain in women who underwent adhesiolysis, EHP-30 score 32.5 (4.4 – 48.8) compared to the controls -5 (-21.3 – 15.0); p < 0.0074 at 6 months. There was also a significant improvement in the EHP-30 social support domain in the adhesiolysis group -12.5 (-71.9 – 0.0) compared to the controls -6.25 (-18.8 – 12.5), p < 0.045 at 3 months although this improvement is not sustained at the 6 months follow up.

There were no complications or adverse events associated with the surgery. Earlier unblinding of any of the patients’ data was not required.

## Discussion

Despite over twenty years of debate over the role of surgical adhesiolysis in women with CPP [11,19], there is no clear clinical consensus as to whether surgically removing adhesions in women with pelvic adhesions and pain is beneficial. This study due to its small sample size unfortunately is at best only able to answer part of this question. A statistically significant improvement of VAS score was shown in women with adhesiolysis compared to those without. Women who underwent adhesiolysis also demonstrated an improvement in physical and emotional well-being.

Modern management strategies of the woman with adhesions and pain range from conservative management of the ‘neurotic’ patient [11] to more extensive surgeries such as hysterectomies [20] and colectomies [21] but the degree and evidence of benefit of these treatments remains variable and questionable. There are two main mechanisms by which adhesions may cause pain. Pelvic/abdominal adhesions contain nerves [22,23] and are attached to pain sensitive mobile structures within the peritoneal cavity. As such, the stimulus generated by stretching and movement may result in pain sensitisation and the anatomical disruption of adhesions may help curtail the sensitisation process.

In theory, women with adhesions removed are more likely to suffer from adhesion reformation, which may result in the lack of improvement or worst pain, and QoL outcomes at follow up. All the participants who underwent adhesiolysis had 4% Icodextrin as an adhesion reduction agent although recent studies have shown this product to be inefficacious [24]; the beneficial effect seen in our outcome measures cannot therefore be attributed solely to the presence or absence of adhesion reformation.

Our study can be criticised in that the study group had a higher adhesion score compared to the controls. Despite this being a randomised controlled trial, due to the small sample size, there was disparity in the baseline characteristics of adhesion score, which may have inadvertently, confounded the results. We observe a large variation in individual scores, denoted by the large interquartile range, which suggests a significant degree of clinical heterogeneity in the study population. However, our study is too small to show effects of surgery on specific types of adhesions or individual surgeon’s effect. The study had a follow up period that spanned a 6-month duration, and this is deemed sufficient to overcome the placebo impact of surgery and large randomised trials have shown little change of pain and QoL scores between the 6 – 12 month follow up mark [13,25].

## Conclusion

CPP is a complex disorder with a high degree of disease burden worldwide [1]. The often-proposed ‘multidisciplinary’ management strategy [10] whilst attractive is financially onerous on health care providers and again lacks evidence-based support for widespread routine practice. Future studies need to take into account the multi-faceted aetiological nature of this disorder, and the effective solutions are likely to be related, not to a single biomolecule or procedure, but to a panel of diagnostic, therapeutic and supportive interventions.

In conclusion, adhesiolysis for women with CPP may improve their pain and QoL in terms of better emotional and physical well being at their 6 month follow up. The potential benefit of adhesiolysis will need to be further evaluated but our study at the least, supports offering adhesiolysis as potentially beneficial treatment modality in some as yet not well characterized instances.

## Competing interest

The authors declared that they have no competing interest.

## Authors’ contribution

YC conceived the idea, is the chief investigator and the main supervisor for the project, was involved with data analysis and wrote the paper. IR analysed and co-wrote the paper. SB and KS conducted the study and co-wrote the paper. WL and TL conducted the study and co-wrote the paper. All authors read and approved the final manuscript.

## Pre-publication history

The pre-publication history for this paper can be accessed here:

http://www.biomedcentral.com/1472-6874/14/36/prepub
